# Oral Manifestation of Autosomal Recessive Congenital Ichthyosis in a 2-Year-Old Patient

**DOI:** 10.1155/2014/483293

**Published:** 2014-06-05

**Authors:** Kavitha Ramar, Sankar Annamalai, V. P. Hariharavel, R. Aravindhan, C. Ganesh, K. Ieshwaryah

**Affiliations:** ^1^Department of Pedodontics and Preventive Dentistry, SRM Kattankulathur Dental College and Hospital, Chennai, India; ^2^Department of Oral Pathology, SRM Kattankulathur Dental College and Hospital, Chennai, India; ^3^Department of Oral Medicine and Radiology, SRM Kattankulathur Dental College and Hospital, Chennai, India

## Abstract

Ichthyosis is a heterogeneous family of hereditary keratinisation disorders mostly characterized by variable erythema of the whole body and different scaling patterns. Although these disorders affect tissues of epidermal origin, there is little evidence regarding the oral and dental manifestations of Lamellar Ichthyosis. A case report of early childhood caries in lamellar ichthyosis is presented and the dental consideration and management is discussed in this paper.

## 1. Introduction

Autosomal recessive congenital ichthyosis (ARCI) is a heterogeneous group of disorders that present at birth with generalized involvement of skin and lack of manifestations in other organ systems. The estimated incidence of lamellar ichthyosis is 1 : 200,000 ± 300,000 [1]. ARCI comprises a group of nonsyndromic ichthyoses that include the phenotype spectrum of classic lamellar ichthyosis and nonbullous congenital ichthyosiform erythroderma, self-healing collodion baby, acral self-healing collodion baby, bathing suit ichthyosis, and harlequin ichthyosis [2]. Mutations in at least 6 different genes (TGM1, ABCA12, NIPAL4 or ichthyin, CYP4F22, ALOX12B, and ALOXE3) are reported to date, but phenotype-genotype correlation has not been fully established [3]. Abnormality in keratinization and exfoliation of the horny cell layer leads to accumulation of hyperkeratotic scales on the skin surface. Symptoms vary from the mildest types such as ichthyosis vulgaris, which may be mistaken for normal dry skin, up to life-threatening conditions such as harlequin ichthyosis. There are several clinical signs of lamellar ichthyosis but there is little information about its oral manifestations and dental management. The uniqueness of the present case is its rareness and suspected correlation of reduced salivary flow and associated early childhood caries.

## 2. Case Report

**Figure 1:**
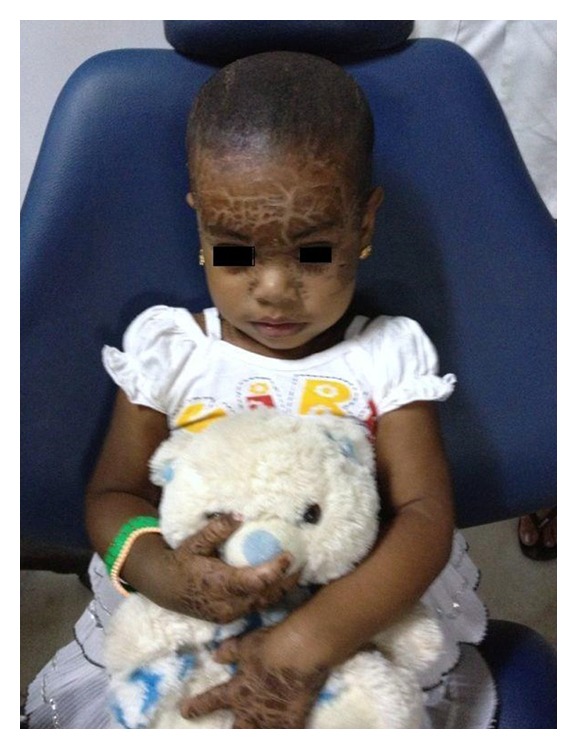
Scaly lesions present in face and hand.

**Figure 2:**
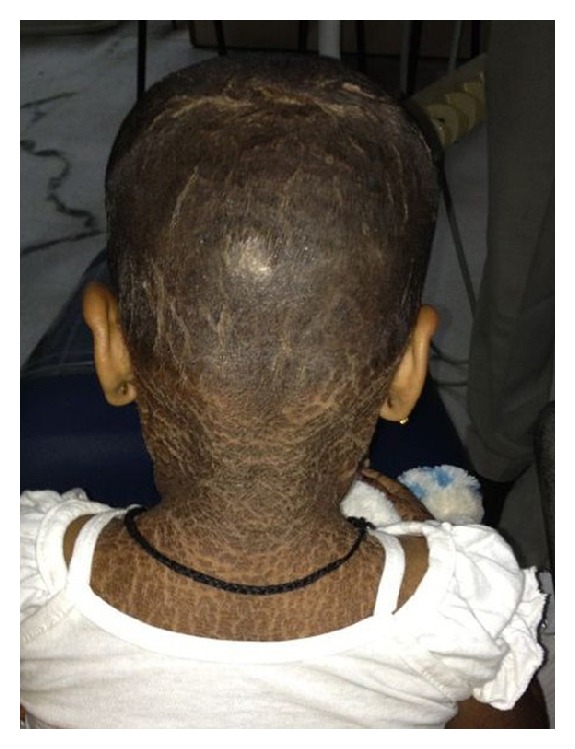
Scaly lesions present in scalp.

**Figure 3:**
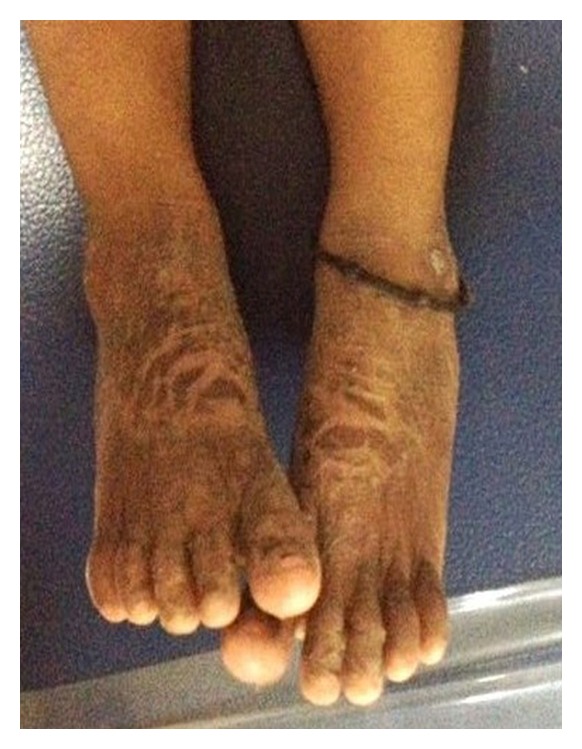
Scaly lesions present in palmoplantar region.

A 2-year-old female patient reported to our department of pedodontia, with the chief complaint of dental caries in front tooth region of the maxilla for the past few months, and revealed no relevant history of pain. The parents also revealed that the patient had dryness of mouth and delayed speech. History reveals that she was born to consanguine parents and she is a “collodion baby” at birth. Few months after birth, the skin showed signs of healing, but later scaly lesions developed in areas like scalp and trunk (Figures 1 and 2), and was diagnosed as Lamellar icthyosis. No ocular, otolaryngeal, and other systemic abnormalities were detected. There was no history of such disease reported in the family. On examination, coarse, dark brown scaly lesions were distributed generally but more prominent in palmoplantar region (Figure 3). Scarring alopecia is also present. On intraoral examination, caries involving cervical region of primary maxillary centrals and laterals was present. The dental management was planned with the restoration of the involved teeth. Regarding dryness of the mouth it was advised to sip water frequently all day long. But it was not possible to quantitatively measure the salivary flow rate as the child's age is too young to cooperate for the salivary flow tests. Topical fluoride application was done. Adequate oral hygiene instructions were given and maintenance protocol was told to the parents and they were also instructed to be on regular follow-up.

## 3. Discussion

Mutation of six different genes has been found to play a role in lamellar ichthyosis. Of these, TGM1 (transglutaminase 1) is thought to be one of the major causes for lamellar ichthyosis. Since the transglutaminase plays a vital role in salivary secretion, we suspect that the improper function of the transglutaminase gene in patients suffering from lamellar ichthyosis might also result in reduced salivary secretion. Hence, xerostomia becomes an important dental manifestation of this disease, with an increased risk of dental caries. Even though we put forward a few points to support our discussion below, we cannot confirm this correlation from a single rare case report alone and need further genetic studies with a larger number of samples.

TGM is an important enzyme in humans, present in liver, epidermis, or blood. It is also found to be present on the surface of oral mucosal cells and is capable of cross-linking pellicle proteins. Present data clearly demonstrates that the TGM contributes significantly to the enzymatic profile of the acquired pellicle layer [4]. Epithelial isoenzyme of transglutaminase covalently cross-links with the salivary components mainly of human parotid saliva. TGM, in association with buccal epithelial cells, may selectively process mucosal pellicle constituents and ultimately affect the mucosal pellicle function [5].

Another point for consideration is the history of delayed speech which was given by the parents. Reduced salivary flow itself can be attributed to the difficulty in normal tongue movements and hence delay in the development of speech. This may not necessarily be a manifestation of lamellar ichthyosis. Healthy babies have also been known to show delayed speech. Isolation of the collodion baby from normal interaction with society may also be a reason for the delayed speech.

With this case report we recommend the necessity to check for salivary flow, advice frequent sipping of water, pit and fissure sealants, topical flourides as a preventive measure in patients with lamellar ichthyosis. If reduced salivary flow rate is confirmed, then active treatment for xerostomia is necessary. Care must also be taken to avoid unnecessary manipulation of the patient's perioral skin during the procedure since it tends to be tender and friable. Also, the possibility for hepatic toxicity is high in patients with lamellar ichthyosis who are on retinoids and hence require a reduction in the dose of local anaesthetic agents during dental treatment. The dentist should therefore be aware of such possible complications and preventive dental care needed. Additional report of cases is needed to confirm the oral manifestations of patients with lamellar ichthyosis and importance should be given to find the salivary flow rate; genetic studies should be supplemented to confirm the correlation.
